# Influence of the Components and Orientation of Hydroxyapatite Fibrous Substrates on Osteoblast Behavior

**DOI:** 10.3390/jfb13040168

**Published:** 2022-09-29

**Authors:** Shiao-Wen Tsai, Yu-Wei Hsu, Whei-Lin Pan, Adhisankar Vadivelmurugan, Pai-An Hwang, Fu-Yin Hsu

**Affiliations:** 1 Department of Biomedical Engineering, Chang Gung University, Taoyuan 33302, Taiwan; 2 Department of Periodontics, Chang Gung Memorial Hospital, Taipei 10507, Taiwan; 3 Department of Bioscience and Biotechnology, National Taiwan Ocean University, Keelung 20224, Taiwan

**Keywords:** hydroxyapatite, strontium-substituted hydroxyapatite, orientation, osteogenesis

## Abstract

Synthetic hydroxyapatite has good biocompatibility, bioactivity and osteoconductive ability because its chemical properties and biological properties are similar to those of bioapatite in bone tissue. Strontium-substituted hydroxyapatite has better degradability than hydroxyapatite and can both promote osteogenesis and inhibit adipogenesis in mesenchymal stem cells. Hence, hydroxyapatite and strontium-substituted hydroxyapatite are widely used as bone graft materials, cell carriers and drug/gene delivery carriers. In addition, osteoblasts cultured on aligned nanofibrous substrates had higher expression of osteogenesis-related genes than did those cultured on random nanofibrous substrates. However, to date, no study has explored the effects of the components and orientation of hydroxyapatite nanofibrous substrates on osteoblastic behavior. In this study, a random hydroxyapatite nanofibrous substrate (R-HANF), a random strontium-substituted hydroxyapatite nanofibrous substrate (R-SrHANF), an aligned hydroxyapatite nanofibrous substrate (A-HANF) and an aligned strontium-substituted hydroxyapatite nanofibrous substrate (A-SrHANF) were successfully fabricated by using the electrospinning technique. The effect of fiber composition on osteoblast-like MG63 cells was assessed by evaluating cell morphology, cell proliferation and osteogenesis-related gene expression. The results showed that MG63 cells cultured on A-SrHANF had higher osteogenesis-related gene expression than those cultured on A-HANF. Additionally, MG63 cells were cultured on R-SrHANF and A-SrHANF to evaluate the effects of fiber orientation on cell behavior. On A-SrHANF, the cells aligned along the direction of the nanofibers, with typical bipolar morphologies, and exhibited higher osteogenesis-related gene expression than cells on R-SrHANF. Hence, the components and orientation of hydroxyapatite nanofibrous substrates are critical parameters affecting the osteogenesis process.

## 1. Introduction

Large bone defects can be caused by trauma, infection, skeletal abnormalities or tumor resection. It is important to develop suitable multifunctional bioactive bone substitutes for treating large bone defects [1,2,3]. Bioactive ceramics, such as bioactive glass, α-tricalcium phosphate, β-tricalcium phosphate, and hydroxyapatite, are widely used as bone graft substitutes for the regeneration of large bone defects. Synthetic hydroxyapatite (HA) has been extensively used for bone grafts because its properties are similar to those of the inorganic component of bone tissue. Strontium-substituted hydroxyapatite (SrHA) has higher solubility than pure HA because the substitution of Ca with Sr induces lattice distortions [4,5]. Ni et al. [6] found that HA doped with 5 and 10 mol% Sr promoted osteoblastic cell differentiation and mineralization. Li et al. [7] demonstrated that Sr ions induced osteoblast differentiation by activating downstream Cbfa1. Some studies have shown that SrHA achieves better bone integration than HA [8,9].

The extracellular matrix (ECM) is a three-dimensional network structure. Electrospinning can be readily utilized to fabricate nanofibers that mimic the hierarchical architecture of the ECM, which can influence cell behaviors, such as attachment, migration, proliferation and differentiation [10,11]. Tsai et al. [12] utilized electrospinning technology to fabricate nanofibrous membranes consisting of carbon nanotubes, polycaprolactone (PCL) and gelatin and found that Schwann cells grown on nanofibrous membranes showed higher levels of NRG1 and P0 protein expression than did those grown on casting membranes. Jose et al. [13] demonstrated that electrospun nanofibers could induce the osteogenesis of mesenchymal stem cells better than flat films.

Surmenev et al. [14] developed PCL nanofibrous scaffolds containing HA or SrHA microparticles by an electrospinning process. The results showed that, compared to PCL scaffolds, scaffolds with SrHA and HA increased the expression of collagen I and facilitated cell mineralization via an increase in osteocalcin production. Han et al. [15] prepared mineralized PLLA nanofibrous membranes with incorporated Sr via an electrochemical deposition method. Compared with pure PLLA and mineralized PLLA nanofibrous membranes, mineralized Sr/PLLA nanofibrous membranes promoted the osteogenic differentiation of bone-marrow-derived mesenchymal stem cells. In our previous study, electrospun hydroxyapatite nanofiber (HANF) and strontium-substituted hydroxyapatite nanofiber (SrHANF) substrates were fabricated, and MG63 osteoblast-like cells exhibited higher expression levels of ALP and osteogenesis-related genes on the SrHANF matrix than on the HANF matrix [16]. 

In addition to the chemical composition of the substrate, interactions between the cells and the substrate topography can lead to specific cell responses to environmental signals and result in different cell functions [17,18]. Bone tissue is a collagen-rich tissue composed of collagen fibers in a parallel arrangement. Zhu et al. [19] fabricated polystyrene nanogrooves that were similar in profile and size to the collagen fibrillar architecture and found that the nanogrooves regulate tissue organization and matrix remodeling in the longitudinal direction to promote the aligned formation of bone tissue. Wang et al. [20] prepared PLLA nanofibrous scaffolds with different fiber orientations to study the effects of fiber orientation on cell behaviors. The results showed that osteoblast-like MG63 cells on the randomly arranged fibrous scaffolds showed irregular shapes, while the cells exhibited shuttle-like shapes on the aligned fibrous scaffold. Guo et al. [21] developed random and aligned polycaprolactone/gelatin fibrous matrices by electrospinning and pointed out that the cell behavior was affected by the fiber orientation. However, to date, there have been no studies evaluating the osteogenic activity of osteoblasts in a fibrous bioceramic matrix. Therefore, the aim of this work was to fabricate random and aligned hydroxyapatite nanofibrous substrates (R-HANF and A-HANF) and random and aligned strontium-substituted hydroxyapatite nanofibrous substrates (R-SrHANF and A-SrHANF). Furthermore, the effect of fiber composition and orientation on the cellular behavior of osteoblast-like MG63 cells was assessed.

## 2. Materials and Methods

### 2.1. Synthesis of the Hydroxyapatite Nanofibrous Substrate

The hydroxyapatite nanofiberous substrate (HANF) and strontium-substituted hy-droxyapatite nanofibrous substrate (SrHANF) were fabricated by using electrospinning technology based on our previous study [16]. Briefly, an ethanol/water solution was used to dissolve hexadecyltrimethylammonium bromide (CTAB, Sigma-Aldrich Chemical Company, St. Louis, MO, USA) and triethyl phosphite (TEP, Merck, Darmstadt, Germany). The precursor solution was prepared by added calcium nitrate tetrahydrate in absolute ethanol and strontium nitrate in deionized water to the above CTAB/TEP solution and then placed in an oven at 60 °C for 12 h. Subsequently, poly(vinyl pyrrolidone) (PVP, MW = 40,000, Sigma-Aldrich Chemical Company, St. Louis, MO, USA) and Pluronic P123 were dissolved in ethanol (99.9%) and then added to the precursor solution. The mixed solution was used to fabricate nonwoven nanofiber via an apparatus (FES-COS, Falco Tech Enterprise Co. Ltd., New Taipei City, Taiwan) under 18 G, 30 kV, 0.508 mL/h and 25 cm for the needle size, spinning voltage potential, spinning flow and distance between the needle and the collector, respectively. Random fibers and aligned fibers were collected by an aluminum-foil-wrapped flat metal plate and a mandrel with a rotation speed of 1500 rpm/min respectively. All of the nonwoven nanofiber structures were calcined at 800 °C to obtain the HANF and SrHANF.

### 2.2. Characterization of HANF and SrHANF

Scanning electron microscopy (SEM, ZEISS ΣIGMA Essential, Oberkochen, Germany) was performed to characterize the morphology of R-HANF, R-SrHANF, A-HANF and A-SrHANF. Substrates were sputter-coated with gold and visualized using SEM with an accelerating voltage of 5 or 10 kV. The average fiber diameters of R-HANF, R-SrHANF, A-HANF and A-SrHANF were analyzed with image analysis software (Image-Pro Express, Media Cybernetics, Rockville, MD, USA) after SEM imaging (Magnification ×5000). The average fiber diameter was calculated from 50 random measurements. In addition, the orientation of the nanofibers was determined by using the OrientationJ plugin for ImageJ (ImageJ software 1.42, National Institutes of Health, Bethesda, MD, USA) on SEM images [22]. The phase compositions of R-HANF, R-SrHANF, A-HANF and A-SrHANF were analyzed by X-ray diffraction with a copper target (XRD, Bruker D2-Phaser, Madison, WI, USA). The scanning region of the diffraction angle (2θ) was from 20° to 70°, with a step size of 0.04°. Spectra were analyzed with DIFFRAC.SUITE EVA software (Bruker v.4.2.) using powder diffraction file (PDF) cards #01-1160-CaO, #85-1108-CaCO_3_, #760694-hydroxyapatite, #89-5631-Sr-substituted hydroxyapatite and #89-5632-Sr-substituted hydroxyapatite as structural models to determine the phase composition.

### 2.3. Cellular Proliferation on HANF and SrHANF Substrates

The R-HANF, R-SrHANF, A-HANF and A-SrHANF substrates (1 cm^2^) were sterilized by ultraviolet light overnight before each cell experiment. Osteoblast-like MG63 cells (5 × 10^4^, BCRC no. 60279, Bioresource Collection and Research Center, Taiwan) were seeded into the substrates in minimum essential medium (MEM) supplemented with antibiotic agent (100 U/mL penicillin and 100 µg/mL streptomycin) and 10% FBS. The proliferative activity was assayed using 3-(4,5-dimethylthiazol-2-yl)-2,5-diphenyltetrazolium bromide (MTT) after 1, 3 and 7 days of incubation.

### 2.4. Cytoskeletal Organization on HANF and SrHANF

The osteoblast-like MG63 cells cultured on the R-HANF, R-SrHANF, A-HANF and A-SrHANF substrates were observed utilizing Alexa Fluor^®^ 488 phalloidin and 2-(4-amidinophenyl)-6-indolecarbamidine (DAPI) staining to visualize the cellular cytoskeletal organization and nuclei, respectively, on day 1. The substrates were fixed with 3.7 wt% paraformaldehyde in phosphate-buffered saline (PBS, 0.02 M pH = 7.4) for 10 min. Subsequently, the substrates were treated with 0.1 wt% Triton X-100 in PBS for 5 min for the permeabilization of cells and then washed thoroughly with PBS. Subsequently, the substrates were incubated with 1 wt% bovine serum albumin for 1 hr to block nonspecific binding sites. After blocking, the substrates were incubated with Alexa Fluor^®^488 phalloidin to stain cellular F-actin and incubated with DAPI to stain the nuclei. Finally, the substrates were observed under a laser scanning confocal microscope (LSCM, Zeiss LSM 780 META, Oberkochen, Germany). The cellular morphology was determined by measuring the length of the long axis of the cell (L) and the maximum width of a cell axis (W) that is perpendicular to the long axis. The aspect ratio (R) was calculated by dividing the L by W.

### 2.5. Gene Expression Analysis Using Real-Time Quantitative Polymerase Chain Reaction (Q-PCR)

After 7 days of culture, 1 mL ice-cold Tri Reagent^®^ was added to the substrate and incubated for 5 min. Subsequently, 100 μL bromochloropropane was added and incubated at room temperature for 15 min. The supernatant was collected after centrifugation at 12,000× *g* for 5 min at 4 °C. Then, 500 μL isopropyl alcohol was added to the supernatant and centrifuged at 12,000× *g* for 10 min at 4 °C to collect the RNA pellet. Then, 1 ml of 75% ethanol solution was added to the RNA pellet and centrifuged at 7500× *g* for 5 min. The precipitate was collected and air-dried. Finally, the precipitate was dissolved in diethylpyrocarbonate water. The concentration and purity of RNA were determined by measuring the absorbance at 260 nm and the ratio of the absorbance at 260 and 280 nm, respectively.

Complementary DNA (cDNA) was synthesized from 5 µg/µL RNA with SuperScript II Reverse Transcriptase (Life Technologies, Carlsbad, CA, USA) according to the manufacturer’s protocol. The expression of the osteoblast mineralization-related genes runt-related transcription factor 2 (RUNX2), collagen type I (COL I), osteopontin (OPN), bone sialoprotein (BSP) and osteocalcin (OCN) was evaluated using real-time quantitative polymerase chain reaction (QPCR). The glyceraldehyde 3-phosphate dehydrogenase (GAPDH) gene expression level was used as an internal control for the normalization of target gene expression. The QPCR primer sequences are shown in Table 1. qPCR was performed on an iQ5 gradient real-time PCR detection system (Bio-Rad, CA, USA) with SYBR Green PCR Master Mix (Protech SA-SQGLR-V2). The following cycling conditions were carried out at 95 °C for 10 min, followed by 40 cycles of 15 s at 95 °C and 60 °C for 60 s. The relative gene expression level was calculated by the 2^−∆∆Ct^ method. MG63 cells cultured in cell culture plates were used as control groups. The fold changes were calculated using the following formulas:

Sample△C_t_ = C_t sample_ − C_t GAPDH_

△△C_t_ = Sample△C_t_ − control△C_t_

The fold change of the sample vs. the control = 2^−∆∆Ct^

### 2.6. Statistical Analyses

Statistical analyses were performed using SPSS v.10. Fiber diameters were analyzed with one-way ANOVA and Tukey’s post-hoc test. Cellular metabolic activity and gene expression were analyzed with the Kruskal–Wallis test, followed by Dunn’s test. A *p* value < 0.05 was considered statistically significant.

## 3. Results and Discussion

### 3.1. Characterization of Fibrous Matrices

**Figure 1:**
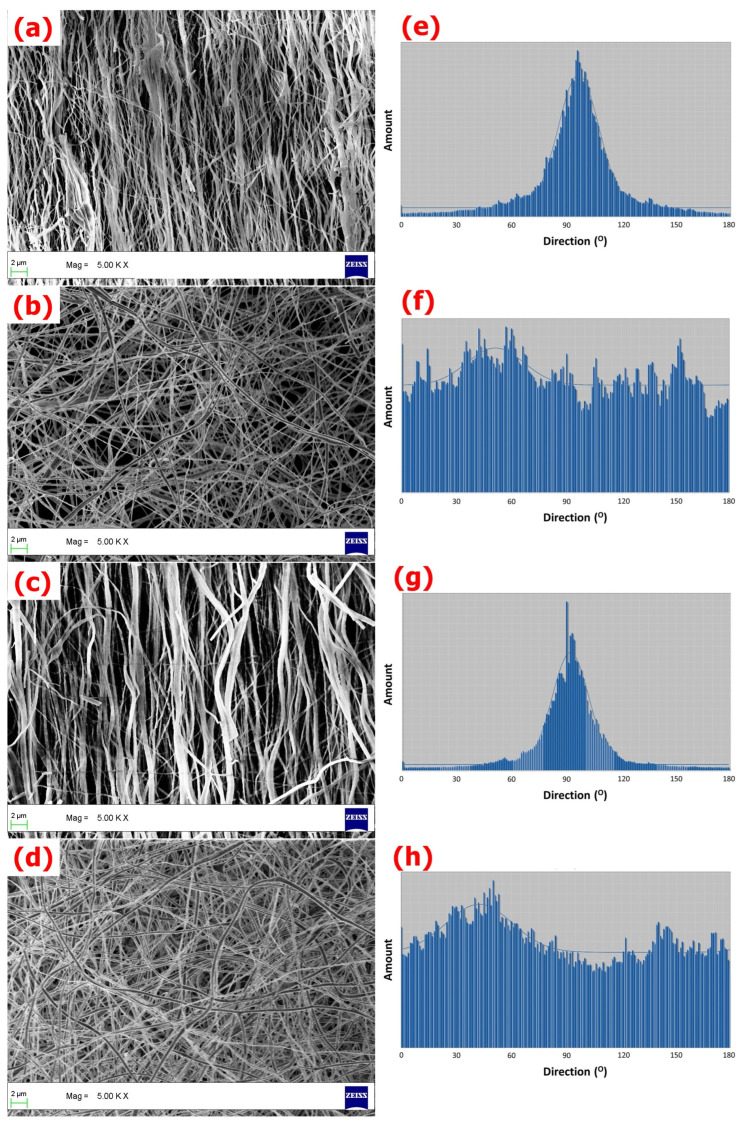
SEM images of (**a**) A-SrHANF, (**b**) R-SrHANF, (**c**) A-HANF and (**d**) R-HANF. The distributions of the orientation angles between the long axes of the nanofibers and their expected directions. (**e**) A-SrHANF, (**f**) R-SrHANF, (**g**) A-HANF and (**h**) R-HANF.

The SEM images of A-SrHANF, R-SrHANF, A-HANF and R-HANF are shown in Figure 1. The average fiber diameters of A-SrHANF, R-SrHANF, A-HANF and R-HANFR-HANF were 555 ± 326 nm, 258 ± 74 nm, 510 ± 244 nm and 287 ± 61 nm, respectively. The microstructure and fiber diameter of fibrous substrates mimic the extracellular matrix (ECM). The fiber diameter was not significantly different between A-HANF and A-SrHANF or R-HANFR-HANF and R-SrHANF. Nevertheless, the average diameter of the aligned nanofibers was significantly larger than that of the random nanofibers. The distribution of the orientations of the nanofibers was determined using OrientationJ software. The full width at half maximum (FWHM) of the curve obtained by fitting the Gaussian distribution was approximately 24.5° and 28.5° for A-HANF and A-SrHANF, respectively.

**Figure 2:**
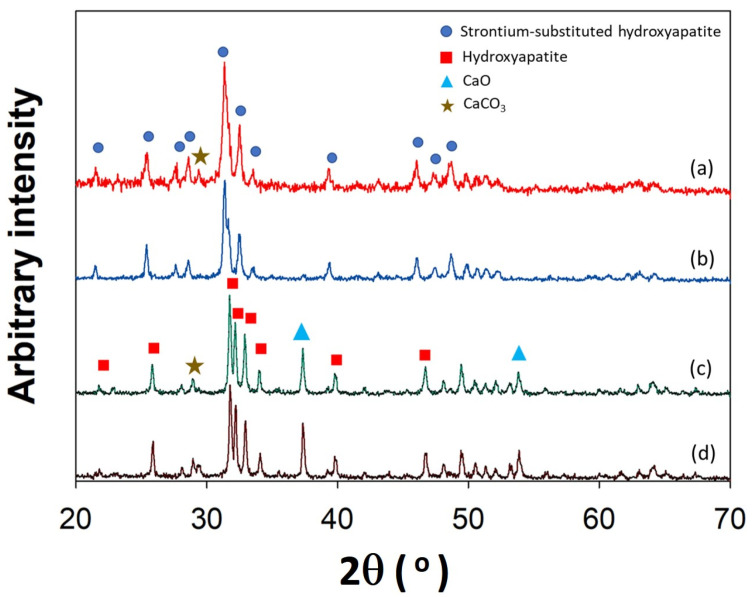
XRD patterns of curve a: A-SrHANF, curve b: R-SrHANF, curve c: A-HANF and curve d: R-HANF.

The crystal structures of A-SrHANF, R-SrHANF, A-HANF and R-HANFR-HANF were examined by wide angle X-ray diffraction (XRD) analysis, as shown in Figure 2. A-HANF and R-HANF both showed several peaks at 2θ = 21.8°, 25.8°, 31.7°, 32.9°, 39.9°, 46.7° and 49.5°, corresponding to hydroxyapatite (PDF card: #89-5632). CaO was characterized by the presence of a peak at 2θ = 37.6° (PDF card #01-1160). In addition, a minor peak was detected at 2θ = 29.6°, which corresponds to CaCO_3_ (PDF card #85-1108). A-SrHANF and R-SrHANF both showed several peaks at 2θ = 21.7°, 25.6°, 31.5°, 31.8°, 32.7°, 39.5°, 46.2° and 48.9°, corresponding to strontium-substituted hydroxyapatite (PDF card #89-5632), and very little CaO and CaCO_3_ was present in A-SrHANF and R-SrHANF. The characterized diffraction peaks of hydroxyapatite in SrHANF shifted to lower 2θ values, which indicates that there is an increase in d-spacing after the incorporation of Sr into hydroxyapatite [23].

### 3.2. Cellular Behavior on the HANF and SrHANF Matrices

**Figure 3:**
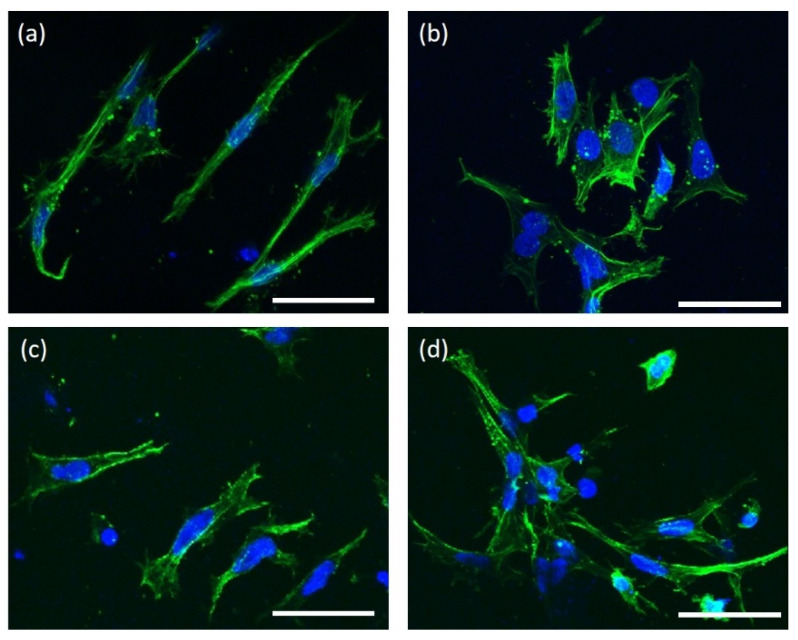
Representative images of MG63 osteoblast-like cells cultured on (**a**) A-SrHANF, (**b**) R-SrHANF, (**c**) A-HANF and (**d**) R-HANF. Cytoskeletal F-actin is stained green with FITC, and cell nuclei are stained blue with DAPI. Scale bar = 50 μm.

Figure 3 shows representative images of MG63 cells cultured on A-SrHANF, R-SrHANF, A-HANF and R-HANF. MG63 cells were stained green using Alexa Fluor® 488 phalloidin to visualize the F-actin component of the cytoskeleton (green fluorescence). The MG63 cells growing on A-SrHANF and A-HANF showed a clear preference to orient themselves parallel to the direction of fiber alignment, while MG63 cells on R-SrHANF and R-HANF showed no preferred orientation. These cells showed significantly larger cell areas on R-SrHANF and R-HANF than on A-SrHANF and A-HANF. Besides, the aspect ratio of MG63 cells on the of A-SrHANF, R-SrHANF, A-HANF and R-HANF were 10.28 ± 4.67, 2.46 ± 1.35, 6.02 ± 0.49 and 1.97 ± 0.69. The MG63 cells on the aligned nanofibrous substrates exhibited higher cell elongation than random nanofibrous substrates. Yin et al. [24] showed that tendon stem cell responded to aligned fiber matrices in a similar manner by elongating and aligning parallel to fiber orientation.

**Figure 4:**
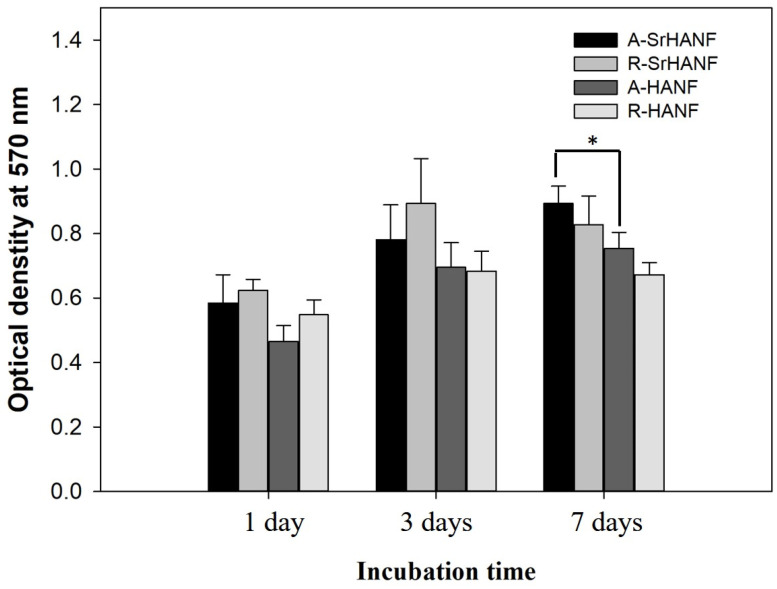
MTT assay quantifying cell proliferation on A-SrHANF, R-SrHANF, A-HANF and R-HANF. The viability of MG63 osteoblast-like cells on various substrates after culturing for up to 7 days. Data are presented as the mean ± SD, n = 3. (*) denotes a significant difference (*p* < 0.05).

Cellular behavior is sensitive to the topography and composition of the substrate. The proliferation rates of MG63 cells on the substrates were analyzed using MTT assays. The MG63 cells on the A-SrHANF showed a significantly higher proliferation rate than those on the A-HANF after 7 days of cell culture (as shown in Figure 4). However, the proliferation rate of MG63 cells after 7 days of cell culture was not significantly different between A-SrHANF and R-SrHANF or A-HANF and R-HANF.

The extracellular matrix of bone consists of type I collagen (~90%), with the remaining ~10% noncollagenous proteins (such as osteocalcin, osteonectin, bone sialoproteins, proteoglycans, osteopontin, fibronectin, bone morphogenic proteins, etc.). Osteoblasts synthesize proteins that are involved in cell adhesion. In this study, MG63 osteoblast-specific gene regulation was observed for the six studied genes: RUNX2, COLI, ALP, OPN, BSP and OCN.

RUNX2 is an essential transcription factor and is involved in many aspects of skeletal development [25]. The DNA-binding sites of RUNX2 in genes encoding major bone matrix proteins, including COLI, ALP, OPN, BSP and OCN, have been identified, and RUNX2 induced the expression of these genes or activated their promoters [26,27,28,29]. During osteoblast differentiation, the expression of RUNX2 is upregulated in preosteoblasts, reaches its maximal level in immature osteoblasts and is downregulated in mature osteoblasts [30].

Fibrous collagen functions as a three-dimensional template to regulate the spatial aspect of mineralization [31]. COLI is regarded as an early-stage marker of osteoblast differentiation during the proliferative and matrix maturation phases [32]. Alkaline phosphatase is widely recognized as an osteoblastic differentiation marker that is expressed during the initial phases of the process. OPN is a phosphorylated glycoprotein and is thought to facilitate the attachment of osteoblasts to the extracellular matrix. OPN is also an early marker for osteoblast differentiation [33]. Boskey et al. [34] found that bone mineral content and size were significantly increased in OPN-knockout mice. OPN plays an essential regulatory role in bone metabolism. However, the precise roles of OPN in osteoblastic functions remain unclear [35].

BSP is an anionic phosphorylated glycoprotein that can bind to the calcium ions of hydroxyapatite with strong affinity [36]. The biological function of BSP is not fully understood. Gordon et al. [37] noted that BSP has a direct effect on osteoblast differentiation and can promote initial mineral crystal formation. Therefore, BSP has long been considered a middle-stage marker of osteoblastic differentiation. OCN is a noncollagenous calcium-binding protein intrinsic to the organic matrix of bones that tightly binds to bioapatite [38]. OCN is essential for the alignment of bioapatite parallel to collagen fibrils. Boskey et al. [39] proposed that osteocalcin might regulate the rate of mineral maturation. Therefore, OCN is considered a late-stage marker of osteogenic differentiation.

**Figure 5:**
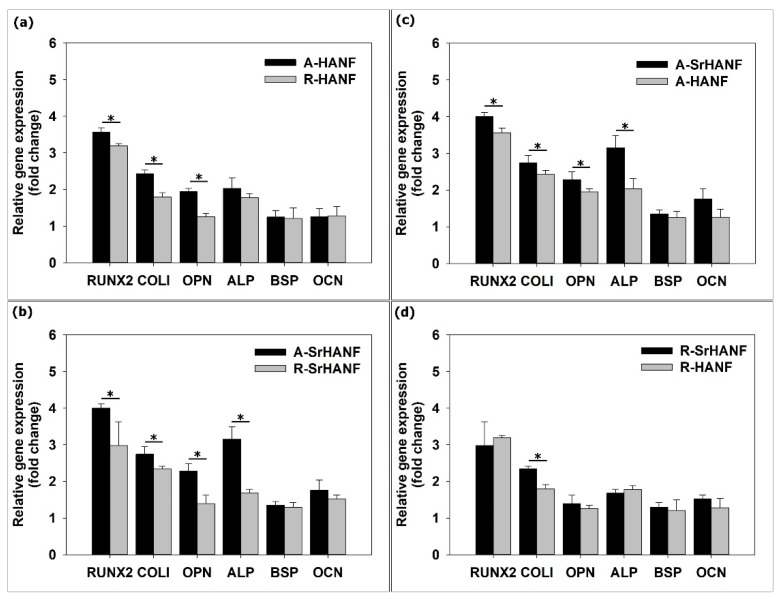
Real-time PCR analyses of bone-associated genes expressed by MG63 osteoblast-like cells on substrates after 3 days of incubation. (**a**) compared aligned fibrous HANF substrates with random ones; (**b**) compared aligned fibrous Sr-HANF substrates with random ones; (**c**) compared aligned HANF and Sr-HANF fibrous substrates; (**d**) compared random HANF and Sr-HANF fi-brous substrates. Gene expression levels after normalization to GAPDH. Data are shown as the fold change relative to the Petri dish after 3 days of incubation. Data are presented as the mean ± SD, n = 3. (*) denotes a significant difference (*p* < 0.05).

**Figure 6:**
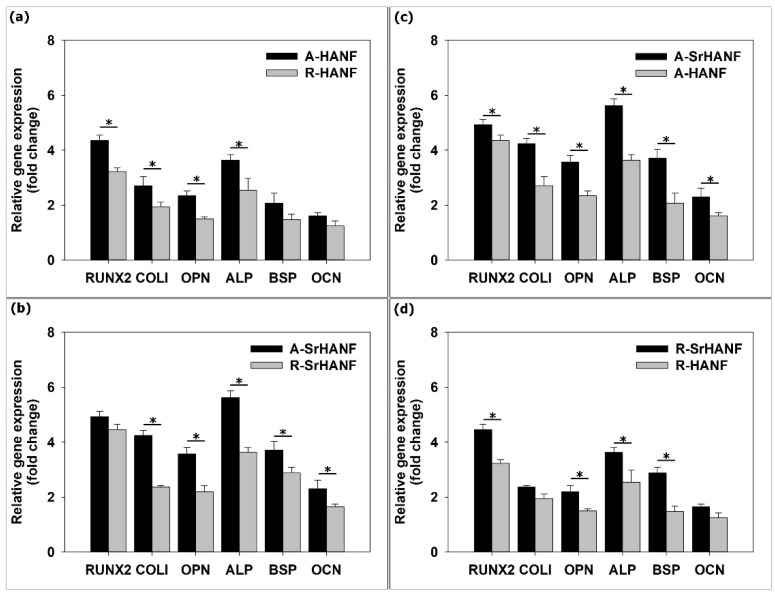
Real-time PCR analyses of bone-associated genes expressed by MG63 osteoblast-like cells on substrates after 7 day of incubation. (**a**) compared aligned fibrous HANF substrates with ran-dom ones; (**b**) compared aligned fibrous Sr-HANF substrates with random ones; (**c**) compared aligned HANF and Sr-HANF fibrous substrates; (**d**) compared random HANF and Sr-HANF fi-brous substrates. Gene expression levels after normalization to GAPDH. Data are shown as the fold change relative to the Petri dish after 3 days of incubation. Data are presented as the mean ± SD, n = 3. (*) denotes a significant difference (*p* < 0.05).

The results showed that the gene expression of RUNX2, COLI and OPN was higher on the aligned fibrous substrates than on the random fibrous substrates on day 3 (Figure 5a,b). Moreover, MG63 cells expressed higher levels of RUNX2, COLI, ALP, OPN, BSP and OCN on the aligned fibrous substrates than on the random fibrous substrates on day 7 (Figure 6a,b). Wang et al. [40] cultured rat mesenchymal stem cells on poly(3-hydroxybutyrate-co-3-hydroxyhexanoate) fibers and found that the expression of the osteogenesis-related gene RUNX2 was higher on aligned fibers than on random fibers. BSP and OCN genes are key regulators of bone matrix mineralization, and their upregulation is expected to promote the mineralization of osteoblastic cells [41]. Chen et al. [42] demonstrated that the alignment of nanofibers induced osteoblastic cells to upregulate RUNX2, COLI, BSP and OCN expression.

In addition, MG63 cells expressed higher levels of RUNX2, COLI, OPN, ALP, BSP and OCN on A-SrHANF than on A-HANF and on R-SrHANF than on R-HANF on day 7 (Figure 6c,d). Strontium is an essential trace element in the human body and has a pronounced ability to stimulate osteoblast differentiation, inhibit osteoclast differentiation and attenuate bone resorption. Kołodziejska et al. [43] noted that strontium can activate the Ras/MAPK/ERK signaling pathway, which leads to the increased activation and phosphorylation of RUNX2 in preosteoblasts. Zhang et al. [44] showed the induction of proliferation, osteogenic differentiation and angiogenic factor expression in MG-63 cells at a certain concentration of strontium, and the stimulatory effect involved the activation of the ERK1/2 and p38 signaling pathways. Gordon et al. [37] demonstrate that increased BSP expression has a direct effect on osteoblast differentiation, as shown by increased RUNX2/Cbfa-1 activity. Therefore, in this study, the results demonstrated that fiber orientation and fiber composition are critical parameters affecting BSP and OCN expression, which reveals the influence of osteoblastic activity and mineralization.

## 4. Conclusions

Bone tissue is mainly composed of collagen and bioapatite. The hierarchical structure and composition of the substrate affect cell attachment, cell extension, migration, proliferation and protein expression on the cell membrane, cytoskeletal activity and other cell behaviors. Compared to polymer nanofibrous substrates, polymer–hydroxyapatite composite nanofibrous substrates fabricated with electrospinning technology have been proven to enhance osteoblastic differentiation. In the present work, we successfully fabricated HANF and SrHANF nanofiber matrices with different orientations via electrospinning. Regardless of components, the proliferation of MG63 cells was higher on aligned matrix than on random ones on day 7. The result is consistent with the conclusion with previous studies that fibrous orientation has effect on cell differentiation. Furthermore, regardless of orientation, MG63 cells on a strontium-containing nanofiber matrix showed higher COLI, RUNX2, OPN, ALP, BSP and OCN expression than on the pure hydroxyapatite matrix on day 7. Together, all results indicated that the composition and orientation have a synergistic effect on cell proliferation and differentiation. The present work showed that the aligned nanofibrous hydroxyapatite substrate containing strontium possessed increased osteogenic potential relative to that of the random nanofibrous hydroxyapatite substrate. Therefore, the aligned strontium-substituted hydroxyapatite nanofibrous substrate is a potential bone substitute for bone repair and regeneration.

## Figures and Tables

**Table 1:** Oligonucleotide primer for PCR amplification.

Gene	Primer Sequence: Sense/Antisense
GAPDH	5′-GAGTCCACTGGCGTCTTCACC-3′
	5′-GACTGTGGTCATGAGTCCTTC-3′
RUNX2	5′-GGAGGGACTATGGCATCAAA-3′
	5′-GCTCGGATCCCAAAAGAAGT-3′
ALP	5′-CACGTCTTCACATTTGGTGG-3′
	5-GCAGTGAAGGGCTTCTTGTC-3′
Collagen Type I	5′-CGGAGGAGAGTCAGGAAG-3′
	5′-CAGCAACACAGTTACACAAG-3′
BSP	5′-TGCCTTGAGCCTGCTTCCT-3′
	5′-CTGAGCAAAATTAAAGCAGTCTTCA-3′
Osteopontin	5′-CTCATTGCTCTCATCATTGG-3′
	5′-AAGCGAGGAGTTGAAATGG-3′
Osteocalcin	5′-CAGCGAGGTAGTGAAGAC-3′
	5′-GCCAACTCGTCACAGTCC -3′

## Data Availability

Not applicable.
